# Bidirectional relationships between increased arterial stiffness and atrial fibrillation

**DOI:** 10.3389/fcvm.2026.1730443

**Published:** 2026-05-28

**Authors:** Yanan Zhao, Zixin Zhang, Qi Qi, Xinyu Wu, Jie Deng, Hongxia Cao, Liying Tian, Aili Zhang, Quanle Han, Shouling Wu, Kangbo Li

**Affiliations:** 1 Catheterization Unit, Tangshan Gongren Hospital, Tangshan, China; 2 School of Clinical Medicine, Hebei Medical University, Shijiazhuang, China; 3 Department of Cardiology, Tangshan Gongren Hospital, Tangshan, China; 4 Department of Urology, Tangshan Gongren Hospital, Tangshan, China; 5 Department of Cardiology, Kailuan General Hospital, Tangshan, China; 6 School of Clinical Medicine, North China University of Science and Technology, Tangshan, China

**Keywords:** arterial stiffness, atrial fibrillation, bidirectional relationships, cohort study, Kailuan study

## Abstract

**Background:**

We investigated the bidirectional relationships between increased arterial stiffness (iAS) and atrial fibrillation (AF) in a cohort study.

**Methods:**

The current study includes two sub-studies. In the first sub-study, the aim was to study the impact of baseline iAS on new-onset AF, 9,072 participants with iAS were propensity-matched with 18,144 non-iAS participants. In the second sub-study, the aim was to study the impact of AF with incident iAS, 193 participants with AF were propensity-matched with 579 non-AF participants. The cumulative incidence of new-onset AF (or iAS) within each group was calculated using the Kaplan–Meier method, with the log-rank test applied to evaluate differences between groups. The incidence rates of AF (or iAS) were determined by dividing the number of events by 1,000 person-years. Cox proportional hazards models were utilized to assess the influence of iAS on the risk of AF and vice versa.

**Results:**

In the first sub-study, during a median follow-up duration of 8.99 years, 96 AF were recorded in the iAS group, whereas 71 new-onset AF were recorded in the non-iAS group. A greater cumulative incidence of new-onset AF was in the iAS group (3.93%) than the non-AF group (2.00%) (*P* < 0.001). Cox regression analysis revealed a positive correlation between iAS and AF [hazard ratio (HR): 6.502, 95% confidence interval (CI): 4.402–9.602, *P* < 0.001)]. In the second sub-study, during a median follow-up duration of 6.49 years, 105 iAS were recorded in the AF group, whereas 273 new-onset iAS were recorded in the non-AF group. A greater cumulative incidence of new-onset iAS was in the AF group (69.31%) than the non-AF group (47.96%) (*P* < 0.001). Cox regression analysis revealed an independent association between AF and iAS (HR: 2.261, 95% CI: 1.636–3.126, *P* < 0.001).

**Conclusion:**

Our findings indicate that there is a bidirectional relationship between iAS and AF. We highlight that preventing iAS is crucial for lowering the risk of AF, while also emphasizing that preventing AF is vital for decreasing the risk of iAS.

## 1 Introduction

Both arterial stiffness (AS), and atrial fibrillation (AF) exhibit similarities in their molecular and pathophysiological mechanisms, with numerous studies highlighting their interdependent relationship (1). Increased AS (iAS) leads to hemodynamic alterations that may play a role in the onset of AF (2). Hemodynamically, a stiffer vascular system results in heightened pulse pressure and an earlier return of the reflected wave from peripheral arteries during left ventricular (LV) systole. This situation causes an increase in central aortic pulse pressure and LV afterload, along with a decrease in ejection fraction, ultimately resulting in LV hypertrophy (3). Additionally, ventricular remodeling elevates left atrial (LA) pressure, which in turn increases LA wall stress and promotes LA remodeling, leading to dilation of the left atrium (4). Various types of atrial remodeling facilitate the onset or persistence of AF by influencing the essential mechanisms of arrhythmia (5). Therefore, the hemodynamic changes associated with iAS may trigger structural and electrical remodeling that contributes to the development of AF (6).

Conversely, AF may play a role in the progression of iAS. While the exact mechanisms through which AF promotes the development of iAS remain unclear, several key risk factors for AF—such as hypertension, diabetes mellitus, and chronic kidney disease—are also linked to the onset of iAS (7–9). AS results from the interplay between hemodynamic forces and the structural and functional changes in blood vessels, which are influenced by the mechanosensitive responses of vascular smooth muscle cells (VSMCs) (10). Notably, numerous studies have indicated that hypertension, diabetes mellitus, and chronic kidney disease stimulate VSMC proliferation through various mechanisms (11–13).

Previous population-based studies have shown that iAS and AF often are associated. There is substantial evidence indicating a link between iAS and AF. Recent research concentrating on iAS and the pre-fibrillatory phases enhance their causal relationship (14). However, the causality association between AF and iAS remains uncertain. To this end, we conducted a bidirectional study to investigate the relationship between iAS and AF in a general population.

## 2 Materials and methods

### 2.1 Participants and study design

The study population was derived from the Kailuan study, a prospective cohort investigation that has included 101,510 participants since its inception in 2006. Each participant completed questionnaires, underwent physical examinations, and participated in laboratory tests, with follow-up assessments conducted every two years. The current study includes two sub-studies. In sub-study one, the aim was to study the impact of baseline AS on the risk of new-onset AF. Subjects with a known history of AF were excluded. The endpoint of sub-study one was new-onset AF. In sub-study two, the aim was to study the impact of AF on the risk of incident iAS. The endpoint of sub-study two was new-onset iAS.

### 2.2 Data collection and definitions

Participants engaged in face-to-face interviews, laboratory evaluations, and physical examinations during the initial survey and at subsequent biennial follow-up visits. Standardized questionnaires were employed to collect information regarding age, gender, sociodemographic characteristics, health-related behaviors, and medical histories. Blood pressure measurements were taken as the average of three readings while participants were seated, utilizing a mercury sphygmomanometer. Blood samples were drawn from the antecubital vein following a 12 h fasting period, and biochemical parameters were analyzed using an auto-analyzer (Hitachi 747, Tokyo, Japan). A higher education level is defined as having completed senior high school or higher. A higher income level is characterized as earning more than 1,000 Chinese yuan per month. Smoking is defined as the consumption of more than one cigarette per day for over a year. Drinking is characterized by an average daily alcohol intake exceeding 100 mL for more than one year. Regular physical activity is defined as engaging in exercise more than three times a week, with each session lasting over 30 min. Snoring status was self-reported by participants, often corroborated by family members, with habitual snoring defined as occurring more than three times per week. The definitions of diabetes, hypertension, and hyperlipidemia were established according to the International Classification of Diseases codes by qualified medical professionals.

### 2.3 Assessment of AS

The BP-203RPE III networked arterial stiffness detector [Omron Healthcare (China) Co., Ltd.] was utilized to measure brachial-ankle pulse wave velocity (baPWV). Participants were requested to lie supine and stay quiet during the assessment. For the upper arms, the airbag logo on each cuff was positioned in line with the brachial artery, ensuring that the lower edge of the cuff was 2 to 3 cm above the cubital fossa. For the lower limbs, the airbag logo was aligned with the medial aspect of the limb, with the cuff's lower edge positioned 1 to 2 cm above the medial malleolus. Electrocardiogram detection devices were affixed to the participants' precordial area and secured to both wrists. Each participant underwent two measurements, and the higher baPWV value from either side was recorded as the final result. A baPWV ≥1800 cm/s was defined as iAS, which has been adopted as the criterion for iAS in previous studies (15–17).

### 2.4 Diagnosis of AF

All participants received 12-lead electrocardiogram (ECG), two experienced cardiologists independently read all ECGs and a team of cardiologists conduct reviews of each patient's medical records. AF was diagnosed according to the diagnostic criteria of latest guideline: A typical 12-lead ECG recording or a single-lead ECG tracing lasting more than 30 s that displays a heart rhythm without identifiable repeating P waves and irregular RR intervals (provided that atrioventricular conduction remains intact) is indicative of clinical AF (18). AF diagnoses were obtained from the discharge registers maintained by the municipal social insurance, which encompasses all participants of the Kailuan study, along with resting ECGs from each follow-up assessment conducted every two years.

### 2.5 Statistical analysis

All statistical analyses were conducted using SAS software (SAS Institute, Inc, Cary, NC; version 9.4). Because advancing age and sex are highly related to both AS and AF (19, 20), a nearest neighbour propensity score matching analysis was performed to match age and sex (caliper: 0.5, standardized mean differences <0.1). Continuous variables were summarized as mean ± standard deviation for normally distributed data and as medians with interquartile ranges for skewed data. Categorical variables were presented as frequencies and percentages. The cumulative incidence of new-onset AF (or iAS) within each group was calculated using the Kaplan–Meier method, with the log-rank test applied to evaluate differences between groups. The incidence rates of AF (or iAS) were determined by dividing the number of events by 1,000 person-years. Cox proportional hazards models were utilized to assess the influence of iAS on the risk of AF and vice versa. All statistical tests were two-tailed, with *P*-values less than 0.05 deemed statistically significant.

## 3 Results

### 3.1 Baseline characteristics

**Figure 1:**
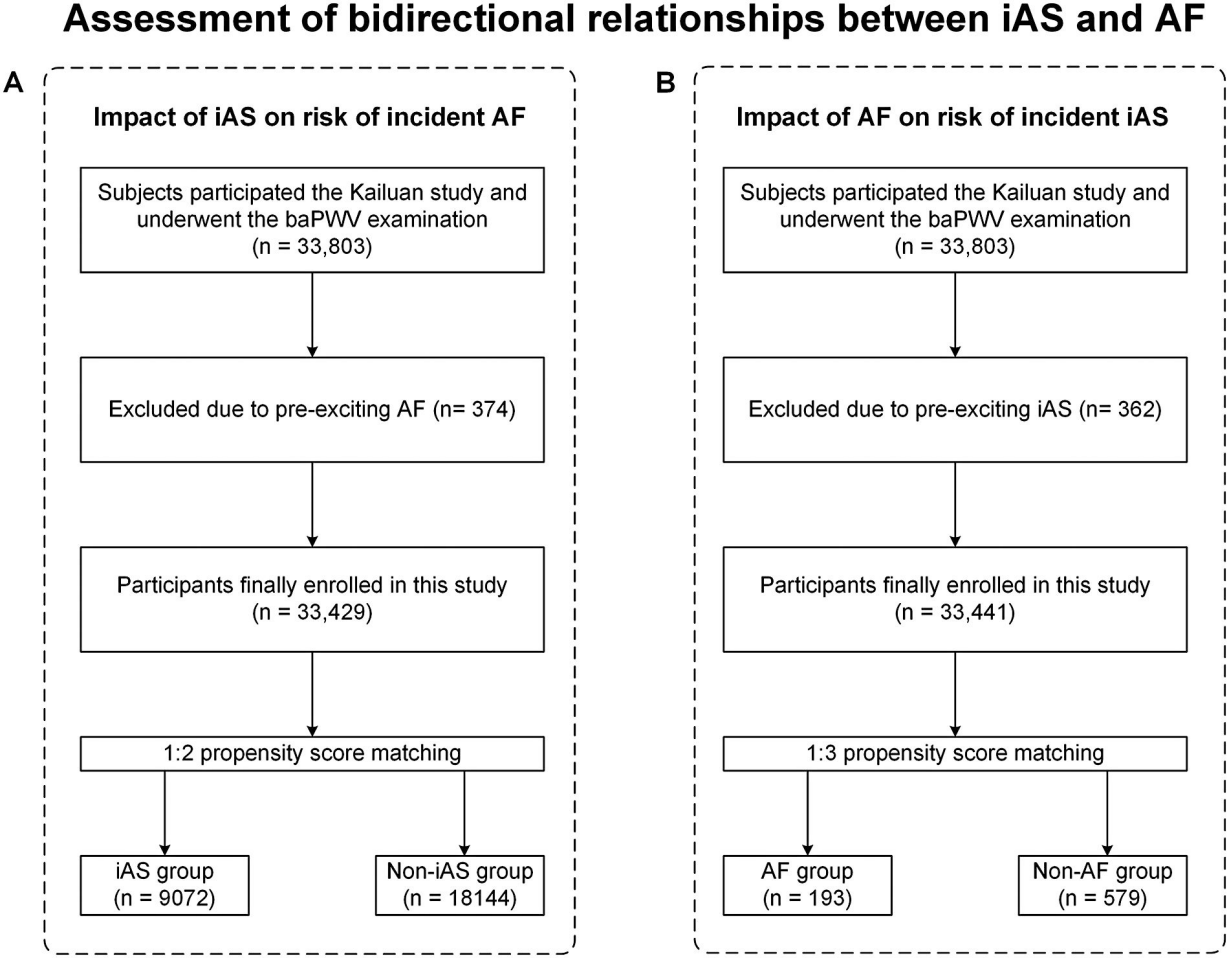
Study flowchart of **(A)** Impact of iAS on risk of incident AF, **(B)** Impact of AF on risk of incident iAS. iAS, increased arterial stiffness; AF, atrial fibrillation.

A total of 33,803 participants were assessed using baPWV since 2010. In the first sub-study, 374 individuals with known AF were excluded. Ultimately, 33,429 participants were included in this study, with 9,072 participants assigned to the iAS group and 24,357 participants assigned to the non-iAS group. Subsequently, 9,072 participants with iAS were propensity-matched with 18,144 participants from the non-iAS group (Figure 1A). Baseline characteristics of the first sub-study are shown in Table 1.

**Table 1:** Baseline characteristics of participants.

	Before propensity matching			After propensity matching		
Variables	AS group (*n* = 9,072)	Non-AS group (*n* = 24,357)	*P* Value	AS group (*n* = 9,072)	Non-AS group(*n* = 18,144)	*P* Value
Age, years	56.36 ± 12.90	45.75 ± 12.28	<0.001	56.36 ± 12.90	46.83 ± 12.99	<0.001
Male, *n* (%)	6,340 (69.89)	17,130 (70.33)	0.733	6,340 (69.89)	12,657 (69.76)	0.998
BMI, kg/m^2^	33.26 ± 14.77	33.98 ± 15.57	0.013	33.26 ± 14.77	24.92 ± 3.56	<0.001
WC, cm	87.22 ± 10.09	85.55 ± 10.55	<0.001	87.22 ± 10.09	86.11 ± 10.46	<0.001
SBP, mmHg	136.89 ± 20.80	125.94 ± 17.33	<0.001	136.89 ± 20.80	127.89 ± 17.67	<0.001
DBP, mmHg	84.34 ± 11.65	80.58 ± 10.48	0.142	84.34 ± 11.65	80.49 ± 10.67	<0.001
FBG, mmol/L	6.11 ± 2.19	5.53 ± 1.98	<0.001	6.11 ± 2.19	5.61 ± 2.14	<0.001
TC, mmol/L	5.12 ± 1.51	4.92 ± 1.63	<0.001	5.12 ± 1.51	4.97 ± 1.67	<0.001
TG, mmol/L	1.38 (0.95–2.15)	1.20 (0.81–1.88)	<0.001	1.38 (0.95–2.15)	1.24 (0.84–1.93)	<0.001
HDL-C, mmol/L	1.53 ± 0.73	1.46 ± 0.76	<0.001	1.53 ± 0.73	1.45 ± 0.61	<0.001
LDL-C, mmol/L	2.73 ± 0.96	2.65 ± 1.21	<0.001	2.73 ± 0.96	2.75 ± 1.10	<0.001
hs-CRP, mg/L	1.40 (0.70–3.00)	1.20 (0.60–2.50)	<0.001	1.40 (0.70–3.00)	1.20 (0.59–2.50)	<0.001
Income more than 1,000 yuan/month, *n* (%)	6,392 (70.46)	17,330 (71.15)	0.465	6,392 (70.46)	13,531 (74.58)	0.668
High school education or above, *n* (%)	3,912 (43.12)	11,622 (47.72)	<0.001	3,912 (43.12)	9,059 (49.93)	<0.001
Exercise regularly, *n* (%)	6,855 (75.56)	18,872 (77.48)	0.001	6,855 (75.56)	14,590 (80.41)	0.039
Smoking, *n* (%)	3,004 (33.11)	8,780 (36.05)	<0.001	3,004 (33.11)	6,657 (36.69)	0.001
Drinking, *n* (%)	4,059 (44.74)	10,877 (44.66)	0.990	4,059 (44.74)	8,222 (45.32)	0.042
Snoring，n (%)	1,929 (21.26)	2,206 (9.06)	<0.001	1,929 (21.26)	1,959 (10.80)	<0.001
Diabetes, *n* (%)	5,343 (58.90)	7,912 (32.48)	<0.001	5,343 (58.90)	6,593 (36.34)	<0.001
Hypertension, *n* (%)	6,224 (68.61)	14,561 (59.78)	<0.001	6,224 (68.61)	11,706 (64.52)	<0.001
Hyperlipemia, *n* (%)	4,133 (45.56)	9,344 (38.36)	<0.001	4,133 (45.56)	7,920 (43.65)	<0.001

AS, arterial stiffness; BMI, body mass index; WC, waist circumference; SBP, systolic blood pressure; DBP, diastolic blood pressure; FBG, fasting blood glucose; TC, total cholesterol; TG, triglycerides; HDL-C, high density lipoprotein cholesterol; LDL-C, low density lipoprotein cholesterol; hs-CRP, high sensitivity C-reactive protein.

In the second sub-study, 362 individuals with known iAS were excluded. Ultimately, 33,441 participants were included in this study, with 193 participants with assigned to the AF group and 33,248 participants assigned to the non-AF group. Subsequently, 193 participants with AF were propensity-matched with 579 participants from the non-AF group (Figure 1B) Baseline characteristics of the second sub-study are shown in Table 2.

**Table 2:** Baseline characteristics of participants.

	Before propensity matching			After propensity matching		
Variables	AF group (*n* = 193)	Non-AF group (*n* = 33 248)	*P* Value	AF group (*n* = 193)	Non-AF group (*n* = 579)	*P* Value
Age, years	66.58 ± 11.54	48.38 ± 13.33	<0.001	66.58 ± 11.54	66.19 ± 11.78	0.921
Male, *n* (%)	152 (78.76)	23,328 (70.16)	0.034	152 (78.76)	477 (82.38)	0.533
BMI, kg/m^2^	26.25 ± 3.71	24.40 ± 3.23	<0.001	26.25 ± 3.71	24.19 ± 3.16	<0.001
WC, cm	89.30 ± 10.49	85.62 ± 10.44	<0.001	89.30 ± 10.49	86.96 ± 10.20	0.023
SBP, mmHg	141.39 ± 20.72	125.48 ± 18.27	<0.001	141.39 ± 20.72	140.89 ± 20.40	0.958
DBP, mmHg	83.60 ± 12.91	81.85 ± 11.06	0.142	83.60 ± 12.91	82.62 ± 11.29	0.601
FBG, mmol/L	6.73 ± 2.62	5.52 ± 1.76	<0.001	6.73 ± 2.62	6.23 ± 2.11	0.028
TC, mmol/L	4.89 ± 1.38	4.88 ± 1.36	0.996	4.89 ± 1.38	5.19 ± 1.11	0.008
TG, mmol/L	1.32 (0.94–1.83)	1.21 (0.83–1.86)	0.128	1.32 (0.94–1.83)	1.35 (0.98–1.93)	0.798
HDL-C, mmol/L	1.43 ± 0.42	1.51 ± 0.67	0.275	1.43 ± 0.42	1.50 ± 0.45	0.139
LDL-C, mmol/L	2.96 ± 1.88	2.57 ± 0.92	<0.001	2.96 ± 1.88	2.87 ± 0.88	0.663
hs-CRP, mg/L	1.69 (0.80–3.21)	1.25 (0.61–2.80)	0.028	1.69 (0.80–3.21)	1.30 (0.60–2.88)	0.074
Income more than 1,000 yuan/month, *n* (%)	131 (67.88)	18,769 (56.45)	0.006	131 (67.88)	429 (74.09)	0.246
High school education or above, *n* (%)	71 (36.79)	14,263 (42.90)	0.232	71 (36.79)	222 (38.34)	0.928
Exercise regularly, *n* (%)	174 (90.16)	21,040 (63.28)	<0.001	174 (90.16)	493 (85.15)	0.213
Smoking, *n* (%)	58 (30.05)	11,655 (35.05)	0.348	58 (30.05)	212 (36.61)	0.254
Drinking, *n* (%)	72 (37.31)	12,561 (37.78)	0.991	72 (37.31)	255 (44.04)	0.261
Snoring，n (%)	108 (55.96)	12,659 (38.07)	<0.001	108 (55.96)	350 (60.45)	0.546
Diabetes, *n* (%)	69 (35.75)	2,981 (8.97)	<0.001	69 (35.75)	150 (25.91)	0.032
Hypertension, *n* (%)	137 (70.98)	11,631 (34.98)	<0.001	137 (70.98)	390 (67.36)	0.644
Hyperlipemia, *n* (%)	131 (67.88)	17,767 (53.44)	<0.001	131 (67.88)	443 (76.51)	0.059

AF, atrial fibrillation; BMI, body mass index; WC, waist circumference; SBP, systolic blood pressure; DBP, diastolic blood pressure; FBG, fasting blood glucose; TC, total cholesterol; TG, triglycerides; HDL-C, high-density lipoprotein cholesterol; LDL-C, low-density lipoprotein cholesterol; hs-CRP, high sensitivity C-reactive protein.

### 3.2 Impact of AS on risk of incident AF

**Figure 2:**
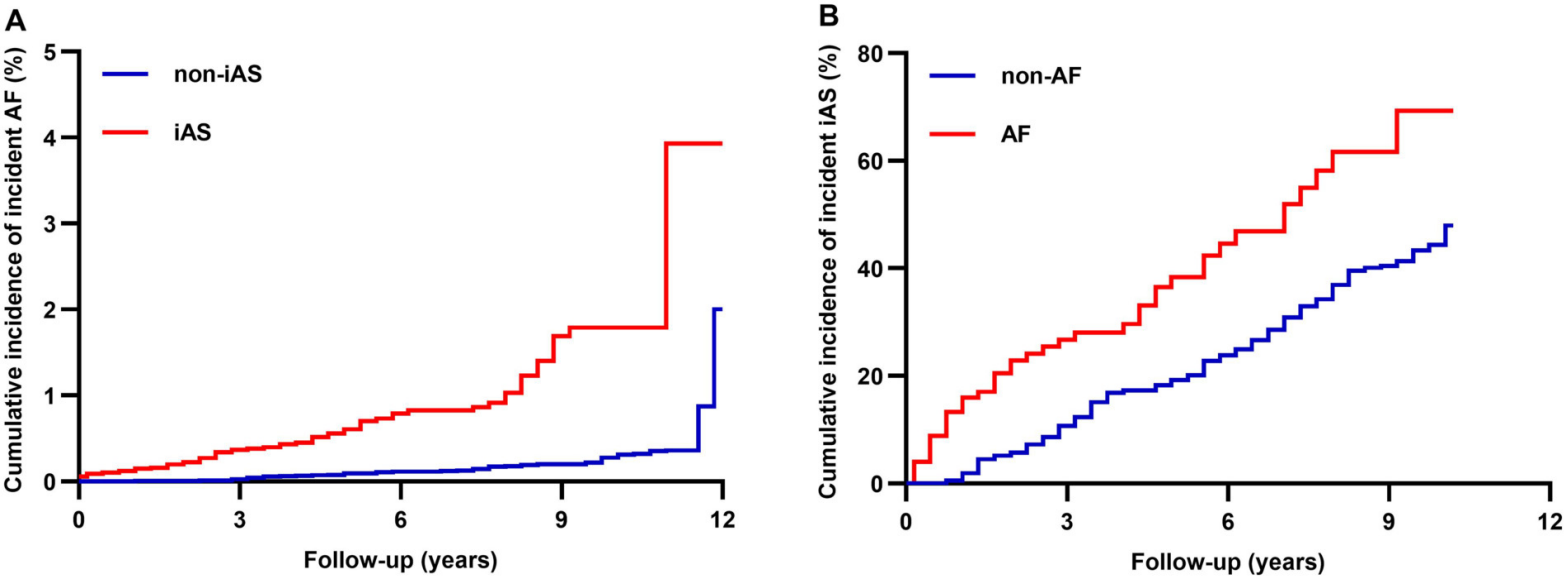
Cumulative incidence of **(A)** AF, **(B)** iAS. iAS, increased arterial stiffness; AF, atrial fibrillation.

During a median follow-up duration of 8.99 years, 96 cases of AF were documented in the iAS group, while 71 cases of AF were observed in the non-iAS group. A higher cumulative incidence of new-onset AF was identified in the iAS group (3.93%) in comparison to the non-iAS group (2.00%) (*P* < 0.001) (Figure 2A). The incidence rates of new-onset AF in the iAS and non-iAS groups were 2.08 and 0.36 per 1,000 person-years, respectively. Univariate Cox regression analysis indicated a positive association between AS and AF [hazard ratio (HR): 6.502, 95% confidence interval (CI): 4.402–9.602]. This relationship remained statistically significant after adjusting age and sex (HR: 4.082, 95% CI: 2.608–6.389), as well after adjusting all variables (HR: 3.996, 95% CI: 2.498–6.391) (Table 3).

**Table 3:** Association of AS with new-onset AF.

Variables	*β*	SE	*χ* ^2^	*P* Value	HR	95% CI	*P* trend
Univariate model							<0.001
AS	1.872	0.199	88.554	<0.001	6.502	4.402–9.602	
Multivariate model 1							<0.001
AS	1.407	0.229	37.888	<0.001	4.082	2.608–6.389	
Age	0.062	0.006	107.115	<0.001	1.064	1.051–1.076	
Sex (men)	0.346	0.242	2.048	<0.001	1.414	0.880–2.272	
Multivariate model 2							<0.001
AS	1.385	0.240	33.423	<0.001	3.996	2.498–6.391	
Age	0.061	0.007	79.593	<0.001	1.063	1.049–1.077	
Sex (men)	0.250	0.281	0.789	0.375	1.284	0.740–2.227	
BMI	0.084	0.026	10.218	0.001	1.088	1.033–1.146	
WC	0.010	0.010	0.861	0.354	1.010	0.989–1.030	
SBP	0.000	0.006	0.002	0.966	1.000	0.987–1.012	
DBP	−0.004	0.010	0.153	0.695	0.996	0.976–1.016	
FBG	0.041	0.027	2.314	0.128	1.042	0.988–1.098	
TC	−0.048	0.079	0.370	0.543	0.953	0.816–1.113	
TG	−0.044	0.064	0.473	0.492	0.957	0.843–1.085	
HDL-C	0.047	0.067	0.490	0.484	1.048	0.919–1.194	
LDL-C	0.092	0.034	7.320	0.007	1.097	1.026–1.173	
hs-CRP	0.011	0.017	0.370	0.543	1.011	0.977–1.046	
Income level	−0.093	0.205	0.208	0.648	0.911	0.609–1.361	
Education level	−0.099	0.197	0.250	0.617	0.906	0.616–1.334	
Exercise level	0.366	0.312	1.377	0.241	1.441	0.783–2.654	
Smoking status	0.006	0.215	0.001	0.978	1.006	0.660–1.534	
Drinking status	−0.170	0.210	0.652	0.419	0.844	0.559–1.274	
History of snoring	0.066	0.195	0.116	0.734	1.069	0.729–1.566	
History of diabetes	0.163	0.237	0.475	0.491	1.177	0.740–1.873	
History of hypertension	0.266	0.261	1.039	0.308	1.304	0.783–2.175	
History of hyperlipemia	0.007	0.239	0.001	0.977	1.007	0.631–1.607	

AS, arterial stiffness; AF, atrial fibrillation; BMI, body mass index; WC, waist circumference; SBP, systolic blood pressure; DBP, diastolic blood pressure; FBG, fasting blood glucose; TC, total cholesterol; TG, triglycerides; HDL-C, high-density lipoprotein cholesterol; LDL-C, low-density lipoprotein cholesterol; hs-CRP, high sensitivity C-reactive protein.

### 3.3 Impact of AF on risk of incident iAS

During a median follow-up period of 6.49 years, 105 cases of iAS were documented in the AF group, while 273 cases of iAS were observed in the non-AF group. A higher cumulative incidence of new-onset iAS was identified in the AF group (69.31%) in comparison to the non-AF group (47.96%) (*P* < 0.001) (Figure 2B). The incidence rates of new-onset iAS in the AF and non-AF groups were 710.2 and 56.12 per 1,000 person-years, respectively. Univariate Cox regression analysis indicated a positive association between AF and iAS (HR: 2.261, 95% CI: 1.636–3.126). This relationship remained statistically significant after adjusting age and sex (HR: 2.587, 95% CI: 1.871–3.576), as well after adjusting all variables (HR: 2.727, 95% CI: 1.938–3.836) (Table 4).

**Table 4:** Association of AF with new-onset iAS.

Variables	*β*	SE	χ^2^	*P* Value	HR	95% CI	*P* trend
Univariate model							<0.001
AF	0.816	0.165	24.397	<0,001	2.261	1.636–3.126	
Multivariate model 1							<0.001
AF	0.950	0.165	33.051	<0,001	2.587	1.871–3.576	
Age	0.061	0.006	121.395	<0,001	1.063	1.051–1.075	
Sex (men)	−0.142	0.144	0.975	0.323	0.868	0.655–1.150	
Multivariate model 2							<0.001
AF	1.003	0.174	33.181	<0,001	2.727	1.938–3.836	
Age	0.061	0.007	79.938	<0,001	1.063	1.048–1.077	
Sex (men)	−0.340	0.171	3.950	0.047	0.712	0.509–0.995	
BMI	−0.058	0.024	5.889	0.015	0.944	0.901–0.989	
WC	0.012	0.007	2.825	0.093	1.012	0.998–1.027	
SBP	0.001	0.004	0.132	0.716	1.001	0.994–1.009	
DBP	0.008	0.006	1.709	0.191	1.008	0.996–1.021	
FBG	0.054	0.034	2.565	0.109	1.056	0.988–1.129	
TC	0.051	0.067	0.594	0.441	1.053	0.924–1.199	
TG	0.056	0.042	1.786	0.181	1.058	0.974–1.149	
HDL-C	−0.011	0.149	0.006	0.939	0.989	0.738–1.325	
LDL-C	−0.105	0.084	1.568	0.211	0.901	0.764–1.061	
hs-CRP	0.018	0.011	2.470	0.116	1.018	0.996–1.041	
Income level	−0.001	0.131	0.000	0.994	0.999	0.773–1.291	
Education level	0.052	0.124	0.172	0.678	1.053	0.825–1.343	
Exercise level	−0.061	0.163	0.140	0.709	0.941	0.684–1.295	
Smoking status	−0.018	0.138	0.018	0.894	0.982	0.749–1.287	
Drinking status	0.041	0.133	0.096	0.757	1.042	0.802–1.354	
History of snoring	−0.104	0.125	0.701	0.403	0.901	0.706–1.150	
History of diabetes	0.235	0.169	1.920	0.166	1.265	0.907–1.763	
History of hypertension	0.551	0.178	9.598	0.002	1.736	1.225–2.461	
History of hyperlipemia	0.025	0.156	0.025	0.875	1.025	0.754–1.393	

AF, atrial fibrillation; iAS, increased arterial stiffness; BMI, body mass index; WC, waist circumference; SBP, systolic blood pressure; DBP, diastolic blood pressure; FBG, fasting blood glucose; TC, total cholesterol; TG, triglycerides; HDL-C, high-density lipoprotein cholesterol; LDL-C, low-density lipoprotein cholesterol; hs-CRP, high sensitivity C-reactive protein.

## 4 Discussion

The present study revealed a bidirectional relationship exists between iAS and AF, with each condition affecting the progression of the other. Indeed, there is substantial evidence indicating a causal relationship between iAS and AF (14). However, research on the impact of AF on iAS are rare. For the first time, we reveal the causality association between AF and iAS in a Chinese cohort. Our findings emphasize the importance of prevention of iAS is critical to reduce the risk of AF; and prevention of AF is essential to reduce the risk of AS. More importantly, AF could be regarded as a risk factor of iAS.

A large number of studies showed that iAS is a predictor of new-onset AF. Chen et al. conducted an analysis involving data of 4,639 participants from the Rotterdam Study. Participants were divided into five groups based on quintiles of aortic PWV. The findings indicated that, in comparison to quintile 1, the adjusted HRs for AF in quintiles 2 through 5 were 0.92 (95% CI: 0.56–1.50), 1.12 (95% CI: 0.70–1.78), 1.54 (95% CI: 0.99–2.41), and 1.47 (95% CI: 0.93–2.32), respectively. When PWV was analyzed as a continuous variable, each standard deviation (3.1 m/s) increase in PWV correlated with a 1.15-fold increase in AF risk (95% CI: 1.03–1.29) (21). In a separate study, Song et al. examined data from 49,872 individuals, identifying 197 cases of AF during an average follow-up period of 6.17 years. Compared to the normal aortic stenosis group, the adjusted HRs of AF in the borderline aortic stenosis and elevated aortic stenosis groups were 1.82 (95% CI: 1.18–2.80) and 2.08 (95% CI: 1.31–3.30), respectively. When baseline baPWV was treated as a continuous variable, each 361 cm/s increase was associated with a 21.7% rise in AF risk (HR: 1.22, 95% CI: 1.08–1.37) (22). Almuwaqqat et al. conducted an analysis involving 3,882 participants who were free of prevalent AF from the Atherosclerosis Risk in Communities Cohort Study. The participants were divided into four groups based on quartiles of carotid femoral PWV (cfPWV). Over a median follow-up period of 5.5 years, 331 new cases of AF were recorded. The study revealed U-shaped relationships between cfPWV levels and the risk of developing AF. The adjusted HR for AF among participants in the first, third, and fourth quartiles were found to be 1.51 (95% CI: 1.07–2.13), 1.58 (95% CI: 1.12–2.21), and 1.58 (95% CI: 1.11–2.25), respectively, when compared to those in the second quartile (23).

Boos et al. conducted a cohort study involving 821 participants. Over a median follow-up period of four years, 75 patients experienced new episodes of AF. The Kaplan–Meier analysis revealed that the cumulative incidence of AF was significantly greater among individuals with a higher baseline ambulatory arterial stiffness index (AASI) compared to those with a lower AASI (*P* = 0.008). Furthermore, when setting AASI as a continuous variable, each standard deviation increases in AASI corresponded to a 1.42-fold increase in the risk of developing AF (95% CI: 1.11–1.82). When setting AASI as a categorical variable, a higher AASI was significantly linked to an elevated risk of AF (HR: 1.69, 95% CI: 1.03–2.79) (24). In addition, Cremer et al. studied 853 hypertensive patients, recording 67 new cases of AF during a 102-month follow-up. They monitored the timing of Korotkoff sounds (QKD), which has been shown to have an inverse relationship with PWV, as a means to assess AS (25). Their findings indicated a significant inverse association between incident AF and QKD (HR: 0.953, 95% CI: 0.93–0.98) (26). Furthermore, Chung et al. analyzed data from 8,048 subjects, measuring the cardio-ankle vascular index (CAVI) to evaluate AS. The multivariate analysis demonstrated a significant association between iAS (CAVI ≥ 8) and the risk of AF, with an odds ratio (OR) of 1.685 (95% CI: 1.908–2.588) (27).

In addition, several studies reported that iAS is associated with AF recurrence. Regarding this, Lau et al. conducted a study involving 68 patients diagnosed with lone AF, measuring pulse pressure to evaluate aortic stiffness. Over an average follow-up period of 2.9 ± 1.4 years, 38 patients experienced a recurrence of AF following their initial catheter ablation. The Kaplan–Meier analysis revealed that patients with a central pulse pressure at or above the 75th percentile had a significantly lower rate of survival free from AF compared to those below the 75th percentile (28). Similarly, Khurram et al. examined 219 patients with AF who were referred for ablation. Among 160 patients, 40 experienced a recurrence of AF after the ablation, with a mean follow-up of 10.4 ± 7.6 months. The Kaplan–Meier analysis indicated that patients with a lower LA stiffness index had a reduced risk of AF recurrence (29). In addition, Shchetynska-Marinova et al. investigated 151 patients with AF who underwent pulmonary vein isolation, measuring aortic distensibility to assess AS. The patients were categorized into four groups based on quartiles of aortic distensibility. The Kaplan–Meier analysis results demonstrated that the cumulative incidence of AF recurrence was significantly higher in the first quartile compared to the fourth quartile (*P* = 0.001) (30). Lastly, Fumagalli et al. studied 31 patients with AF who underwent electrical cardioversion. Logistic regression analysis indicated that for every one-unit rise in CAVI, the risk of AF during the control visit was 2.31 times greater (95% CI: 1.01–5.25) (31).

Most cross-sectional studies reported that AS is significantly higher in patients with AF than in controls, however, a causal relationship has not been confirmed. Miyoshi et al. conducted a study involving 181 outpatients, comprising 91 individuals diagnosed with AF and 90 without. They measured the CAVI to evaluate AS. The findings revealed that the CAVI was significantly elevated in patients with AF compared to those without (9.0 ± 1.0 vs. 8.7 ± 0.8, *P* < 0.01) (32). In a similar investigation, Pauklin et al. examined 76 patients with paroxysmal or persistent AF and 75 healthy controls. Their results demonstrated that patients with AF exhibited a higher PWV than the healthy controls (8.0 m/s vs. 7.2 m/s, *P* < 0.001) (33).

Mendelian randomization studies have recently emerged as a more reliable method compared to traditional observational studies, as they effectively reduce confounding factors and the risk of reverse causation (34). In a study conducted by Zekavat et al., the causal link between iAS and AF was examined through Mendelian randomization. The findings revealed a significant correlation between genetically elevated ASI and AF, with an OR of 1.8 per standard deviation increase in ASI phenotype (95% CI: 1.4, 2.2; *P* = 3.1 × 10^−7^). Conversely, no significant association was found in another analysis (95% CI: −0.007, 0.009; *P* = 0.89). Therefore, the authors concluded that a genetic tendency towards higher ASI is unidirectionally linked to an increased risk of both incident and prevalent AF (35).

As mentioned earlier, both AS and AF share similarities in their molecular and pathophysiological mechanisms (1). Consequently, certain conditions, such as heart failure with preserved ejection fraction (HFpEF), are significantly associated with both AS and AF. The interplay between AS, AF, and HFpEF is of great importance and warrants discussion. On the one hand, AS is increasingly acknowledged as a vital element in the pathophysiology of HFpEF, influencing diagnosis, management, and prognosis. As a defining characteristic of vascular aging, AS leads to an increased afterload on the LV, resulting in diastolic dysfunction, which is a key aspect of HFpEF (36).

On the other hand, HFpEF and AF are becoming more prevalent, and exhibit similar clinical characteristics. Both HFpEF and AF share common risk factors and comorbidities, such as hypertension, aging, obesity, and obstructive sleep apnea syndrome, which predispose individuals to both conditions concurrently (37). Moreover, LA enlargement in HFpEF is recognized as a significant proarrhythmic substrate linked to atrial fibrosis (38). The abnormal distribution of gap junctions and the loss of cell-to-cell coupling in fibrotic areas contribute to electrical remodeling, heightened atrial refractoriness, and the onset of AF (39). Conversely, since AF itself leads to LA dilation, impaired atrial function, and atrial fibrosis, it may directly contribute to the development of HFpEF (40). AF is also correlated with LV myocardial fibrosis, which subsequently plays a role in diastolic dysfunction and HFpEF (41, 42). Furthermore, neurohormonal pathways may represent another mechanism through which AF induces HFpEF. Persistently elevated atrial filling pressures activate neurohormonal pathways; these mechanisms encourage myocardial fibrosis, heightened extracellular matrix accumulation, and cardiomyocyte hypertrophy, resulting in ventricular remodeling (43, 44).

### 4.1 Limitation

Our study has several limitations. First, while PWV is a useful indicator of AS, the reliable clinical data under the circumstances of different types arrhythmia are still lacking, it may not be entirely reliable in individuals with AF due to the irregular and variable nature of the heart rhythm in AF. In fact, current guidelines typically regard arrhythmia as a condition where PWV measurements should not be conducted (45, 46). Second, AF is classified as paroxysmal-, persistent-, long-standing persistent-, and permanent AF based on its duration. But, the detailed types of AF were not recorded in our database. AF frequently presents without symptoms, which can result in it going undetected until complications arise or even after they have developed. The estimated undiagnosed AF has been derived from patient screening, ranging from 1% to 2% of the general population (47). Nevertheless, AF diagnoses were obtained from the discharge registers maintained by the municipal social insurance and resting ECGs during follow-up biennially in the present study. In addition, the prevalence of undiagnosed AF was not estimated, which may lead potential grouping error in the present study. Third, although all baseline variables were adjusted in the Cox proportional hazards models, some critical medication history (e.g., anticoagulant drugs, antiarrhythmic drugs) were not considered. Residual confounding occurs when those confounders have not been adequately adjusted for in present the analysis. Furthermore, despite this bidirectional relationship study was scientifically designed, reverse causality is a major source of bias in observational studies, particularly in chronic disease research. Therefore, it warrants further research (e.g., Mendelian randomization study) in this topic.

## 5 Conclusion

A bidirectional relationship was found to exist between iAS and AF, with each condition influencing the development of the other. Preventing iAS is crucial for lowering the risk of AF, while also emphasizing that preventing AF is vital for decreasing the risk of iAS.

## Data Availability

The raw data supporting the conclusions of this article will be made available by the authors, without undue reservation.
